# Relationship between Early Childhood Caries and Prolonged Coughing Episodes in a Cohort of Cambodian Children

**DOI:** 10.3390/ijerph191912842

**Published:** 2022-10-07

**Authors:** Noureen Chowdhury, Bathsheba Turton, Tepirou Chher, Sithan Hak, Gabriela Hondru, Karen Sokal-Gutierrez

**Affiliations:** 1 Faculty of Dentistry, University of Puthisastra, Phnom Penh 120211, Cambodia; 2 Office of Global and Population Health, Henry M. Goldman School of Dental Medicine, Boston University, Bostson, MA 02118, USA; 3 Oral Health Bureau, Department of Preventive Medicine, Ministry of Health, Phnom Penh 12151, Cambodia; 4 UNICEF Cambodia, Phnom Penh 120211, Cambodia; 5 School of Public Health, University of California, Berkeley, CA 94704, USA

**Keywords:** early childhood caries, prolonged coughing, respiratory tract infections, Cambodia, modifiable risk factor

## Abstract

Studies have shown an association between Early Childhood Caries (ECC) and respiratory infections; however, most have been cross-sectional, and all have been in high-income countries. Inverse probability treatment weighting (IPTW) was applied to longitudinal data from the Cambodia Health and Nutrition Monitoring Study. An analytical sample of 1703 Cambodian children between 1- and 4-years old was used to examine the effect of caries incidence (ECC Activity) on the odds of a child subsequently experiencing an episode of prolonged coughing (>14 days) over the subsequent 18 m. ECC activity occurred among 523 children (30.7%) while prolonged coughing was observed among 235 children (13.8%). ECC activity increased the risk of prolonged coughing (RR 1.23; 95% CI 0.95, 1.58; Average treatment effect = 3%). Follow-up investigations are justified in order to examine whether ECC may be a modifiable risk factor for prevention of respiratory illness among young children.

## 1. Introduction

Early Childhood Caries (ECC), or tooth decay, is the most prevalent disease among children. In 2015 untreated ECC affected 573 million children globally [1,2]. ECC is considered a chronic and non-communicable disease, driven by the interaction between the oral bacterial biofilm and dietary free sugars. Exposure of the bacterial biofilm to dietary sugars creates an imbalance whereby acid producing and aciduric bacteria dominate and the environment at the surface of the tooth becomes acidic. Such an acidic environment leads to a loss of mineral from the tooth surface and subsequently cavitated carious lesions [3]. When an active carious lesion is left untreated it can have adverse short- and long-term impacts on a child’s health, growth, and development [3]. Untreated caries commonly causes mouth pain, reduced chewing capacity, and loss of sleep and appetite, and can result in compromised nutritional status, overall health, and quality of life [4,5,6]. The 74th World Health Assembly’s resolution to integrate oral health care into universal health care highlights the importance of oral health to overall health and wellbeing globally [7,8].

Oral disease also leads to considerable social, health, and economic burdens [9], disproportionately affecting socioeconomically-disadvantaged populations [7,10]. In Cambodia, dental caries, chronic malnutrition, and respiratory infections are among the most prevalent childhood diseases. The consequences of these childhood diseases not only influence negative child growth but also burden the national economy [11], with a consequence modelling estimating a $400 million annual burden due to malnutrition in Cambodia [12]. The 2011 Cambodian National Oral Health Survey found that by age six, 93% of children experienced dental caries, including 86% with dental abscesses, and 99% of carious lesions were untreated [13,14]. More recent studies reported that by age 3, an estimated five out of six children had one of more deep carious lesions and one in five had a tooth which has been abscessed [14], and children with untreated dental caries and abscesses had less favorable ponderal growth [15].

Recent studies in high-income countries have found an association between dental caries and respiratory infections in early childhood [16,17]. According to the WHO Maternal Child Epidemiology Estimation in 2018, pneumonia accounted for 14% of all deaths in children under 5 years in Cambodia [18]. The 2014 Cambodian Demographic and Health Survey (CDHS) found 6% of children under the age of five experienced illness accompanied by coughing and short, rapid breathing in the two weeks prior to the survey. This information was collected based on the mother’s perception and reporting. The key findings also indicated that nearly 7 out of 10 children with acute respiratory illness symptoms required medical assistance at a health facility or from a provider [19].

A recent study suggested that the pathway between dental caries and respiratory illness is through migration or aspiration of oral pathogens into the respiratory tract leading to chronic bronchitis or pneumonia and/or reactive airways. Respiratory illness is among the major causes of morbidity and mortality in young children, with pneumonia as the main cause of mortality reported for children under five years old [20]. Pneumonia causes nearly 1.3 million deaths per year, majority of which are preventable with asthma remaining as the most common non-communicable disease in children [21]. Early childhood respiratory illness (similarly to ECC) can contribute to poor sleep, loss of appetite, increased need for medical attention, and use of medications. Those things impact school attendance, growth and development, and overall quality of life, with common risk factors being environmental exposures and poor nutrition.

There is a need for further investigation into the phenomena of chronic oral infection and inflammation and the subsequent impact on child respiratory health, especially in low- and middle-income countries. The aim of this paper was to examine the relationship of caries incidence (ECC activity) and respiratory symptoms (prolonged coughing), using data from a cohort of Cambodian children.

## 2. Materials and Methods

This secondary analysis explored the relationship between ECC activity and parental report of prolonged coughing; reporting followed STROBE guidelines. Data were sourced from the Cambodia Health and Nutrition Monitoring Study (CAHENMS), a collaborative study among the Ministry of Health, UNICEF Cambodia, and the French National Institute for Sustainable Development (IRD) to provide enhanced monitoring of health and nutrition in six districts of Cambodia. The study provided a unique opportunity to observe numerous health indicators, including respiratory symptoms. The CAHENMS study was initiated in July 2016 and the dental module was added during June 2017 to run annually from there. The CAHENMS involved collection of longitudinal data on health, nutrition, and diet of pregnant and lactating women and their children (<5 years of age), collected across the three Cambodian provinces which did not have access to fluoridated water sources: Phnom Penh, Ratinakiri, and Kratie [22].

Ratanakiri is the most north-eastern province of Cambodia; the population density is low and villages are scattered. Kratie is another of the northeastern provinces and it borders Vietnam to the South. Data were collected from the two central administrative districts of the province (Chet Borei and Krong Kratie). Kratie province displays two distinct regions whereby the northeast part of the province is a forested plateau suitable for cattle rearing and plantations. The Southwest is a wet plain fertilized by the Mekong River, where smallholder farmers successfully grow rice, corn, beans, and horticulture products. In both agro-ecological regions, large amounts of land have been turned to economic concession zones. Phnom Penh province houses the capital and the peri-urban district of Russei Kheav in Phnom Penh city was the third site for recruiting participants. The population in Russei Kheav district is of mixed ethnical origin, including a large community of Cham. This urban poor population was included in order to capture data from urban poor households which comprise around one quarter of residents in Phnom Penh’s residents.

These provinces were identified by UNICEF for Enhanced Health Monitoring over a 3-year period from 2016. The protocol for primary data collection was reviewed and approved by the National Ethics Committee for Health Research, Ministry of Health, Cambodia (NECHR 117). Informed consent was obtained from the caregivers in writing at the time of recruitment and again verbally prior to caregiver participation in follow-up interviews and child intra-oral examinations. The protocol for the secondary data analysis of deidentified data was reviewed by the University of Puthisastra (Phnom Penh, Cambodia) research committee.

### 2.1. Intra-Oral Examination

The CAHENMS involved eight data collection teams in a field environment. These teams were supplemented by a two-person dental team with one calibrated examiner and one trained assistant. The dental team was co-ordinated through the existing logistical processes put in place through UNICEF and also through the Oral Health Bureau of the Ministry of Health. Eight senior dental students who served as examiners as part of the dental teams underwent calibration training until all achieved a kappa score above 0.9 for the Criteria for ECC lesion Classification Index, indicating near-perfect agreement. Children were examined in a supine position with a mouth mirror and illumination from a hand-held torch. Appropriate infection control procedures were performed for management of non-aerosol procedures. Data were collected on caries status using the three-stage ECC classification index [23] and the Pulpally involved Ulcerated Fistula Abscess index [24], as described in previous publications [14].

### 2.2. Questionnaire

Trained personnel administered validated questionnaires to the caregiver of each participating child. There were two interview formats: An in-person interview (referred to as ‘the full questionnaire’) conducted at the time of clinical examinations at each follow-up contact, including questions about socio-demographics, diet, and hygiene; and a phone interview (referred to as ‘the morbidity questionnaire’) every 4-to 6-weeks asking whether the child had any illness and for how many days the child was sick as consistent with Demographic and Health Survey methodologies [25,26].

Data were collected and managed using the CAHENMS study database system, the “KoBo Toolbox”. Data were entered directly onto tablets for uploading at a location where there was WiFi access. For the present analysis, data were drawn from the KoBo Toolbox database in a Microsoft Excel spreadsheet format prior to being transferred for analysis.

### 2.3. Analytical Sample

**Figure 1:**
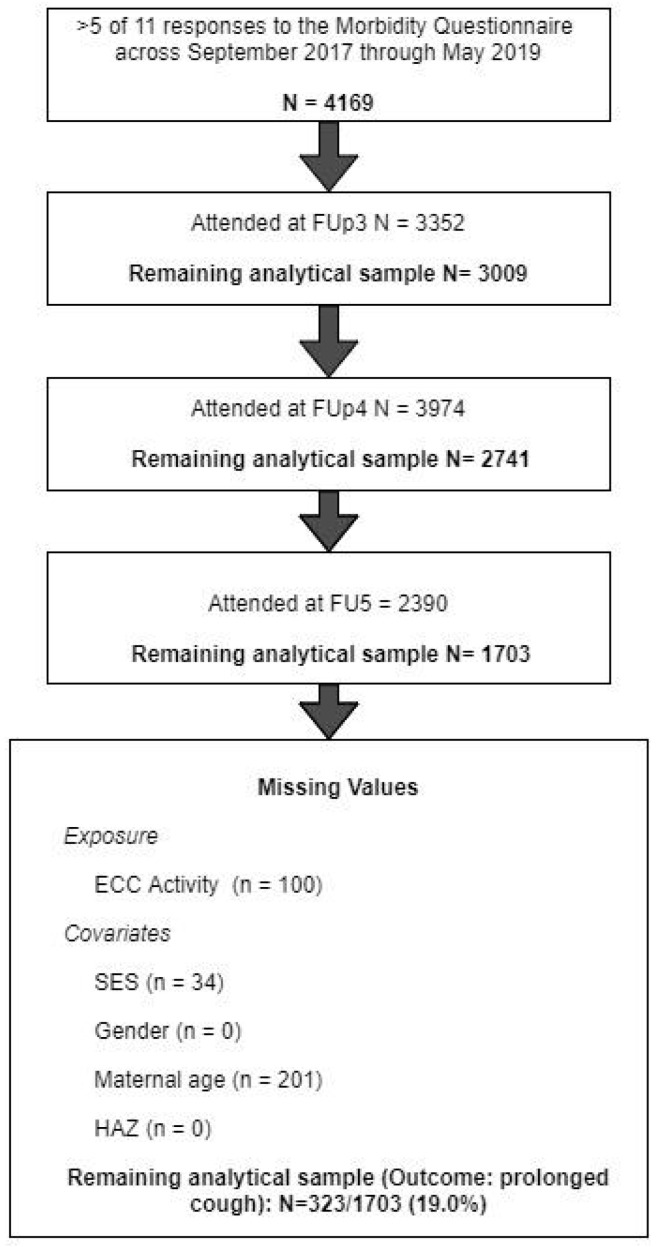
Analytical Sample. Abbreviations; FUp = Follow-up; N = Number of participants; SES = Socioeconomic status; HAZ = Height for Age Z-score; 14d = 14 days.

The current analysis used a subsample of participants who met the following inclusion criteria; they were present at FUp 3, 4, and 5, and they responded to the morbidity questionnaire on 5 or more occasions from when dental data were collected (*n* = 1703). Figure 1 presents an overview of the analytical sample (Supplementary Materials).

### 2.4. Exposure

The exposure was ‘ECC activity’, defined according to the incidence of one or more new cavitated lesions between FUp4 and FUp5.

### 2.5. Covariates

**Figure 2:**
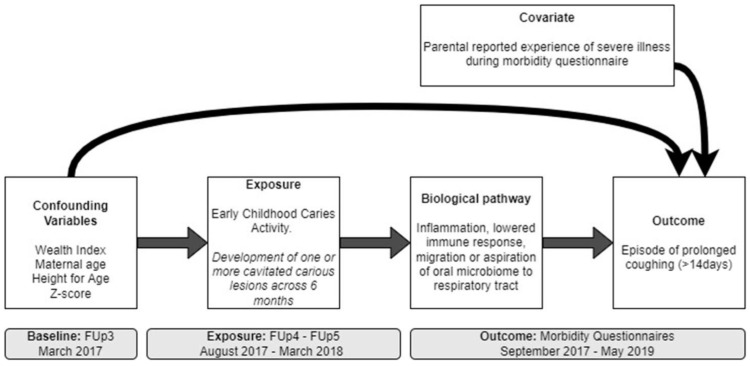
Directed acyclic graph for the relationship between caries exposure and episodes of prolonged cough. Abbreviations: FUp = Follow-up.

Covariates were selected based on the literature and included stunting at FUp3, parental report of severe illness other than coughing that required a visit to the doctor, and social determinants of ECC and common diseases. The considered determinants were the child’s sex (Male/Female), location (Kratie/Ratanakiri/Phnom Penh), household socio-economic strata (SES), maternal age (number of years), and maternal education (None or informal/Primary education/Secondary education). The SES indicator was generated by using a procedure consistent with the Demographic and Health Survey recommendations. The SES was calculated as the five quintiles of wealth index computed based on Principal Component Analysis (PCA). The indicators used in the PCA are the household ownership of generator, telephone, radio, TV, PC, bike, motorbike, boat, refrigerator, employment status, the type of materials used for the house roofing and walls [27]. The hypothesized theoretical relationship between ECC, covariates, and coughing episodes lasting more than 14 days (prolonged coughing) outcomes is presented in a directed acyclic graph (DAG) in Figure 2.

### 2.6. Outcome

Parents who reported one or more episodes of prolonged coughing (more than 14 days) when questioned using the morbidity questionnaire [25,26].

### 2.7. Statistical Analysis

STATA 16 was used to perform regression and inverse probability treatment weighting analysis (IPTW). This analytical approach is a standardization-based approach applied for confounding control that maximizes exchangeability between the exposed and unexposed groups by achieving conditional randomisation [28]. It maximizes exchangeability by creating a pseudo-population that was balanced upon measured covariates in the analysis. A number of sensitivity analyses were carried out. First, diagnostics were performed to examine how much of covariate imbalance between exposure groups was corrected for by reweighting after IPTW estimations. Next, analysis was repeated among an exposure-free cohort at baseline, whereby those who had one or more caries lesions at baseline were excluded. Finally, E-values were calculated to determine the minimum strength of association on the risk ratio scale, which an unmeasured confounder would need to have with both the exposure and the outcome to fully explain the specific exposure-outcome association [29]. *p*-values were considered as significant at values below 0.05.

## 3. Results

There was no difference in follow-up or missing data by maternal characteristics, age, or the presence of one or more episodes of parental reported ‘serious illness’; however, caregivers in higher SES strata were less likely to have responded 5 or more times to the morbidity questionnaire, and were 5–10% more likely to have one or more missing data points (Tables S1 and S2).

Most children were below the two years of age at baseline (mean age = 19.4 months old; SD 9.3 months) and most came from a low socioeconomic background where mothers had an average of six years of education. One in five children had a parental report of a significant episode of illness requiring a visit to the doctor. Nearly one-third (30.7%) of children in the sample demonstrated ECC activity. One in seven children (13.8%) subsequently experienced prolonged cough over an 18-month observation period. Those in the highest and the lowest SES strata were less likely to have a parental report of prolonged coughing. Children from higher SES strata were more likely to develop one or more new cavitated carious lesions and more likely to incur pulpally-involved carious lesions (Table 1).

Estimates from the IPTW analysis showed that the standardized mean probability of having a prolonged cough was 3% (95% CI −0.01, 0.07) higher among children who developed one or more new caries lesions (Table 2). This translated to a relative risk of 1.23 (95% OR 0.95, 1.58). While the estimates did include some uncertainty, the size and directionality of the relationship was consistent and skewed towards the increased chance of prolonged coughing among children who had ECC activity. The E-value was E = 1.76 for the estimate and E = 1.29 for the CI. In the sensitivity analysis where the analytical sample was limited to children who did not have any caries lesions present at FUp4 (Table S4, Supplementary Materials), the effect of the relationship between new ECC lesions and prolonged coughing remained, although the degree of uncertainty increased with a broader confidence interval (RR = 1.19; 95% CI = 0.87, 1.62; Table S3).

## 4. Discussion

This secondary analysis found that children who had ECC activity were more likely to have a subsequent prolonged coughing episode. This study was the first to examine the relationship between dental caries and respiratory symptoms among an LMIC population and the first to apply IPTW to examine the relationship. Overall, exposure to ECC activity leads to a 23% increase in the risk of parental report of prolonged coughing. This difference could be clinically relevant if prevention and treatment of dental caries were deemed to be a modifiable risk factor for the prevention of respiratory illness.

In this sample, one in three children experienced active caries and subsequent increased risk of developing prolonged coughing episodes. This finding is consistent with a US-based study examining the relationship between ECC and Upper Respiratory Tract Infection (URI) where there was a 1.6 times greater risk [16]. Another study from Hong Kong found a similar relationship between caries among children aged 4 and suggested that the oral cavity acts as a reservoir for opportunistic pathogens of the respiratory tract [17]. The relationship between oral health and respiratory tract infections among other age groups has also been explored. One study investigating the association of dental caries as a risk factor of lower respiratory infections in young adults, found it to be the highest in those with 10 or more filled teeth [30].

The biologically plausible pathways for causation that have been suggested are (1) the suppression of the immune system due to the chronic inflammation caused by the pathological biofilm present during the caries process and during dental infections and (2) migration and/or aspiration of specific microbes from the oral cavity and into the respiratory tract [17]. Among older adults, particularly those in long-term care, decayed teeth and cariogenic bacteria in the plaque and saliva have been associated with a higher risk of pneumonia [31,32,33,34]. Bacteriological assays in children could help to understand the biological mechanism and explain the observed effects.

The key limitation of the study was that the outcome measure was a parental report of their child’s prolonged cough and not a medically-diagnosed respiratory illness. For this reason, it is not known if the prolonged cough was caused by an upper or lower respiratory tract infection, allergies, reactive airways, or other cause, which limits the pathophysiologic understanding of the relationship between ECC and respiratory symptoms. Another limitation is the issue of recall bias in the outcome measure, while that was minimized by having more than 10 contact points at which the question was asked it is still possible that under-estimation inaccuracies may exist. Given the limitations of the dataset and the possibility of measurement error on the outcome, follow-up studies with larger sample sizes and longer observation periods would be helpful. Variables on water, sanitation, and hand hygiene were not included as covariates since the investigative approach was to use a minimum and guaranteed set of covariates in order to avoid creating bias through over-controlling for confounders. The same logic applies when justifying the use of SES as a key risk factor for dental caries rather than including other known and subordinate mediating covariates. Although these limitations exist, the E-values suggest that unmeasured confounding would need to have a large (OR > 1.7) effect in addition to the effect accounted for by SES if it were to negate the observed effect of ECC activity on parental reported prolonged coughing.

The strength of this analysis relates to the unique data set with the ability to measure caries incidence prior to the prolonged coughing outcome, and the ability to robustly control for confounders in appropriate temporal relationship. The effect of ECC on prolonged coughing appeared to be overestimated by the logistic regression modeling and so the IPTW analysis was essential for establishing maximum exchangeability among groups and minimizing the effects of selection bias. The effect was present among the caries-free-at-baseline cohort and the E-values were greater than RR related to other covariates. While a small degree of uncertainty remains, the findings provide justification for further investigation of the effect of caries on respiratory conditions, especially in children from low- and middle-income countries.

The implications of the findings in the context of the COVID-19 pandemic are unclear. After data collection on this study was completed, the pandemic spread of SARS-CoV-2 increased the proportion of children with prolonged respiratory symptoms [35]. In addition, disruption of economic and health systems has had adverse impacts on child health. Early estimates of the COVID-19 pandemic suggested a 10% increase in the prevalence of wasting malnutrition over 6 months [36] and in parallel, a reduction in access to oral health care has led to increased prevalence and severity of untreated caries [37]. In the context of COVID-19, and other future respiratory disease pandemics, there appears to be an even greater need to explore whether preventing and treating ECC could help prevent child respiratory symptoms, especially in LMIC where the morbidity and mortality is greatest.

## 5. Conclusions

This study was the first to examine the relationship between ECC activity and prolonged coughing in an LMIC environment. Exposure to active ECC was associated with an increased risk of developing a prolonged cough which parents reported as lasting more than 14 days. Further epidemiological and microbiological studies are required in order to better understand the clinical implications of this finding.

## Figures and Tables

**Table 1:** Sociodemographic and clinical characteristics by exposure to ECC activity ^a^.

	ECC Activity				Prolonged Coughing			
	Develops Dentine Lesions		Develops Pulpally Involved Lesions		Parental Report of Coughing Lasting >14d		All Participants	
	N/Mean	Row %/SD	N/Mean	Row %/SD	N/Mean	Row %/SD	N/Mean	Column %/SD
Sex								
Male	189	21.6	75	8.6	121	13.9	873	51.3
Female	171	20.6	88	10.6	114	13.7	830	48.7
Mean Age in months at FUp3 ^b^	16.5	8.8	24.3	8.7	18.3	10.1	19.4	9.3
SES strata ^b^								
Lowest	52	15.8	16	4.8	46	13.9	330	19.8
Low	67	17.6	27	7.1	47	20.3	381	22.8
Middle	94	23.0	46	11.3	53	22.9	408	24.4
High	62	23.3	35	13.2	47	20.3	266	15.9
Highest	77	27.1	36	12.7	38	16.5	284	17.0
Maternal Characteristics								
Maternal age (years)	27.9	5.8	28.0	5.4	27.3	6.0	27.2	5.9
Years of maternal education	6.1	3.6	6.4	3.6	5.4	3.5	5.9	3.4
Parental report of child illness								
No serious illness	337	21.3	149	9.4	208	13.2^b^	1580	92.8
One or more episodes of serious illness	23	18.7	14	11.4	27	22.0	123	7.2
Prolonged coughing								
No prolonged cough	300	20.4	138	9.4	-	-	1468	86.2
≥1 episode of prolonged coughing	60	25.5	25	10.6	-	-	235	13.8
Total	360	21.1	163	9.6	235	13.8	1703	100.0

^a^ Abbreviations: SES = Socio-economic status; ECC = Early Childhood Caries; ECC Activity = presence of lesion progression, either developing a dentine lesion or developing a pulpally involved lesion; FUp3 = Follow-up 3; N = Number of individuals in a group; SD = Standard deviation; Significant *p*-values indicated separately ^b^ *p*-value = <0.01; chi-squared test for the difference in SES strata by caries progression.

**Table 2:** Odds ratios, average treatment effects, and risk ratios for the relationship between caries progression and significant coughing.

	Coefficient	95% Confidence Interval
Logistic regression model for Cough >14d ^a^		
Adjusted log regression	1.34	0.99, 1.80
IPTW modeling ^a^		
Average treatment effects Coeff. (95% CI)	0.03	−0.01, 0.07
Risk Ratios	1.23	0.95, 1.58
E-values ^c^		
Treatment effect	1.76	
Confidence interval	1.29	

^a^ Number of observations = 1703; Number exposed = 523; Models controlled for the following confounders: for sex, age, maternal education, Socioeconomic Status, and Height for age Z-score and the report of significant illness as a covariate. ^c^ E-value calculated as RR + SQRT(RR x RR - 1)).

## Data Availability

Data may be available from corresponding author upon reasonable request.

## Supplementary Materials

The following supporting information can be downloaded at: https://www.mdpi.com/article/10.3390/ijerph191912842/s1 Table S1: Comparison of characteristics with complete and incomplete data; Table S2: Comparison of characteristics by attrition; Table S3: Standardised raw treatment values for prolonged coughing by caries progression; Table S4: Odds ratios, Average treatment effects and Risk Ratios for the relationship between caries progression and significant coughing where there was no caries at baseline.
