# Supplementary material for: Relationship between Early Childhood Caries and Prolonged Coughing Episodes in a Cohort of Cambodian Children
Source: Int J Environ Res Public Health. 2022 Oct 7;19(19):12842. doi: 10.3390/ijerph191912842 (PMC9566348; doi:10.3390/ijerph191912842)
Supplement: Supplementary file 1 [file ijerph-19-12842-s001.zip › ijerph-1903581-supplementary.pdf]

Supplementary Materials

Table S1. - Comparison of characteristics with complete and incomplete data [a]

	Full case N = 1380		Missing data N = 323		
	n/mean	Column %/sd	n/mean	Column %/sd	P-Value [b]
Sex					
Male	694	50.3	179	55.4	0.055
Female	686	49.7	144	44.6	
SES strata					
Lowest	295	21.4	35	12.1	<0.001
Low	338	24.5	43	14.9	
Middle	338	24.5	70	24.2	
High	205	14.9	61	21.1	
Highest	204	14.8	80	27.7	
Maternal Characteristics					
Maternal age (years)	27.3	6.2	27.0	3.8	0.333
Years of maternal education	5.8	3.3	6.6	3.9	<0.001
No serious illness	1275	94.2	305	94.4	0.123
One or more episodes of serious illness [c]	105	7.6	18	5.6	
Total	1380	81.0 [d]	323	19.0 [d]	

[a] Abbreviations: SES = Socio-economic status; ECC = Early Childhood Caries; ECC Activity = presence of lesion progression, either developing a dentine lesion or developing a pulpally involved lesion; FUp3 = Follow-up 3; N = Number of individuals in a group; SD = Standard deviation

[b] P-value for differences in proportions among groups was generated using the chi squared test. P-value for differences in means among groups was calculated using the standard t-test.

[c] Serious illness reported during morbidity questionnaires defined as an episode of illness where the parent took the child to see a doctor.

[d] Percentage presented is row percentage rather than column percentage as suggested in the column heading

Table S2. - Comparison of characteristics by attrition

	< 5 MU datapoints N = 343		≥ 5 MU datapoints N= 3009		Overall N = 3352	
	N/mean	Row %/sd	N/mean	Row %/sd	N/mean	Column %/sd
Sex						
Male	164	9.7	1528	90.3	1692	50.5
Female	179	10.8	1481	89.2	1660	49.5
SES strata[b]						
Lowest	70	10.6	593	89.4	663	20.1
Low	95	12.7	652	87.3	747	22.7
Middle	84	10.8	691	89.2	775	23.5
High	43	8.5	463	91.5	506	15.4
Highest	47	7.8	553	92.2	600	18.2
Parental illness report						
No serious illness	322	10.2	2820	89.8	3142	93.7
One or more episodes of serious illness	21	10.0	189	90.0	210	6.3
Maternal Characteristics						
Maternal age (years)	27.0	6.1	27.4	6.2	27.2	6.2
Years of maternal education[c]	5.0	3.7	5.8	3.6	5.7	3.6
Total	343	10.2	3009	89.8	3352	100.0

[a] Abbreviations: MU = morbidity questionnaire; SES = Socio-economic status; N = Number of individuals in a group; SD = Standard deviation

[b]Data missing from 61 participants P-value = 0.030; chi-squared test for difference in Morbidity data points by SES strata

[c]P-value = <0.001; Fishers T-test for difference in years of maternal education by number of Morbidity data points.

Table S3. - Standardised raw treatment values for prolonged coughing by caries progression

	Standardized differences		Variance ratio	
	Raw	Weighted	Raw	Weighted
SES strata				
Lowest[a]				
Low	-0.15	<0.001	0.81	1.00
Medium	0.10	<0.001	1.12	1.00
High	0.12	<0.001	1.24	1.00
Highest	0.18	<0.001	1.35	1.00
Parental illness report				
No serious illness[a]				
Had an episode of serious illness	-0.02	-0.01	0.95	0.98
Sex				
Male[a]				
Female	<0.00	-0.01	1.00	1.00
Height for age z-score at baseline	0.12	<0.001	1.14	1.11
Mothers age	0.05	<0.001	0.95	1.00

[a] Reference category

Table S4. - Odds ratios, Average treatment effects and Risk Ratios for the relationship between caries progression and significant coughing where there was no caries at baseline [a].

	Coefficient	95% CI
Logistic regression model for Cough >14d		
Adjusted log regression	1.30	0.90, 1.88
IPW modelling		
Average treatment effects Coeff. (95% CI)	0.03	-0.02, 0.08
Risk Ratios	1.19	0.87, 1.62
E-values [b]		
Treatment effect	1.67	
Confidence interval	1.15	

[a] Number of observations = 1058; Number exposed = 323; Models controlled for the following confounders: for sex, age, maternal education, Socioeconomic Status, and Height for age Z-score and the report of significant illness as a covariate.

[c] E-value calculated as RR+SQRT(RR*RR-1))
